# Effects of Motivational and Behavioral Factors on Job Productivity: An Empirical Investigation from Academic Librarians in Pakistan

**DOI:** 10.3390/bs13010041

**Published:** 2023-01-03

**Authors:** Khurram Shahzad, Shakeel Ahmad Khan, Abid Iqbal, Omer Shabbir

**Affiliations:** 1 Department of Library, Senior Librarian, Government College University Lahore, Lahore 54000, Pakistan; 2 Department of Information Management, Islamia University of Bahawalpur, Bahawalpur 63100, Pakistan; 3 Central Library, Prince Sultan University, Riyadh 11586, Saudi Arabia; 4 Human Resource Department, Prince Sultan University, Riyadh 11586, Saudi Arabia

**Keywords:** motivational factors, job performance, relation of motivation with job productivity, library professionals, librarians

## Abstract

The major objective of the current study was to find out the impact of motivational factors on the job outcomes of librarians working in HEC-recognized university libraries in Pakistan. A survey research method followed by predictive correlational design was applied to test the constructed hypotheses in this study. The population of the study was library professionals working in the university libraries of Lahore, Pakistan. There were 13 public sector universities and 21 private sector universities. The census sampling technique was used to collect data from the respondents of the 34 universities. Data were collected with the help of a questionnaire. Out of 225 respondents, 189 completed questionnaires were received. Hence, the response rate was 84%. The gathered data were analyzed through SPSS software. Descriptive and inferential statistical tests were applied to find out the impact of motivational and behavioral factors on the job outcomes of information professionals. The findings of the study showed that different types of motivation influenced information professionals to carry out innovative and value-added services in the workplace. Rewards, a sense of honor, an amicable work environment, and autonomy were the key categories of motivation that encouraged information professionals to undertake efficient job performance. Recommendations provided through a framework based on the findings of the study will prove to be a benchmark for policymakers, human resource managers, and heads of institutions in order to formulate such techniques that might motivate information professionals for the implementation of user-centric services.

## 1. Introduction

Motivation is known as the reason for the initiation of a behavior that stimulates the meeting of organizational objectives [1]. It involves an employee working to achieve organizational goals as a result of their initiative [2]. It is a revolutionary drive that urges a professional working in an academic setting to produce satisfactory results and productive performance [3].

The theory of motivation involves the attitude of individuals that is shaped in response to extrinsic and intrinsic factors. Intrinsic motivation takes place due to the personal interest of an individual towards a task [4], whereas extrinsic motivation occurs as the result of a response to an external reward [5]. Extrinsic motivation is significant in terms of increasing job performance [6] and producing outcomes [7].

The autonomy of library professionals in the workplace has a strong relationship with effective job performance. Different motivational factors including good remuneration, sufficient resources, the implementation of emerging tools, recreational opportunities, and pleasant mutual relations in the workplace provide job satisfaction to university library professionals [8]. Innovative changes have taken place in libraries due to the revolution in information technology, and university library professionals in present times are expected to initiate value-added services to provide the community with the latest methods, and this mission may be accomplished if library workforces are motivated. The workplace of academic librarians boosts their morale and motivates them towards adopting a dynamic approach to the provision of user services [9].

Job satisfaction is the most crucial element for executing the functions of an organization effectively and efficiently [10,11,12,13,14]. For the attainment of job performance from the academic library workforce, employees’ needs should be satisfied and they should be motivated to work collectively for the accomplishment of the common goals of the institutions [15]. Several factors, including a reasonable salary, a pleasant work environment, appreciation from employers for completed tasks, promotions, the consistent provision of incentives, empowerment, authority, and amicable relations among colleagues, play a fruitful role in enhancing the work output and the satisfaction level of academic library employees [16].

A university library is a microcosm of the university; therefore, internally motivated staff need to be recruited so that the organization can achieve its objectives [17]. Both intrinsic and extrinsic factors drive academic library staff to provide the best results in all tasks that are assigned by the top management of the universities [18]. Transformational leadership in academic libraries proves to be highly significant in improving the motivational level of library professionals, which contributes to positive job performance [19].

## 2. Statement of the Problem

The library profession is very challenging and continuously evolving. It demands the timely motivation of library professionals to achieve organizational objectives. This study intends to measure the impact of motivational factors on the job performance of academic library professionals in Pakistan. With advancements in technological tools, library services are changing rapidly. Therefore, library professionals may not satisfy the information and research needs of their users unless they are motivated through different incentives and rewards. User-centric services may be provided if the library staff is internally motivated and satisfied with their work conditions. For the attainment of exceptional job performance, staff motivation is of great significance. Innovative library services may not be provided to the end-users without motivated and skilled library staff.

## 3. Literature Review

### 3.1. Motivation

The term “motivation” originated from the Latin word “movere”. It means to go ahead or to meet a specific need [20]. It is related to the ability that causes dynamic changes in an individual’s attitude [21]. It relates to the efforts of an individual to show a willingness to work towards a task as a result of specific incentives provided to them [22]. It is also known as inspiration, which is provided to an individual by an organization for the delivery of efficient results.

Motivation is willpower that motivates library professionals to develop high goals for growing personally and professionally [23]. Motivation aims to influence the library workforce through certain incentives for carrying out job functions enthusiastically. Motivated employees are more likely to play a leading role in the attainment of organizational goals [24]. Motivated library staff working in academic libraries may produce better results and work output through value-added services [25].

### 3.2. Employee Motivation in the Academic Setting

Motivational factors have a powerful influence on employees’ minds as they better understand their role in the organization. They work with high spirits for bringing innovation during different operations at the workplace [26]. Internally and externally motivated employees continuously enhance their professional skills and align their personal goals with organizational goals to show maximum efficiency [27]. Employees’ needs, psychological traits, and expectations should be considered carefully by employers for receiving the best output and work results [28]. The performance of employees usually depends upon motivation and satisfaction with their job [29].

Empowerment is a great source of motivation that provides authority to employees for working independently and performing efficiently [30]. Good working conditions have a direct impact on employees’ motivation towards their job as a positive spirit of teamwork is created [31,32]. The level of commitment is increased if the staff are well motivated [33].

### 3.3. Job Satisfaction in Academic Libraries

Job satisfaction refers to employees’ expectations for benefits from the job [34]. A positive mental state as a result of a pleasant work experience is known as job satisfaction [35]. Job satisfaction provides a sense of security and protection to the workforce of an organization [36,37,38].

Independence and authority to make decisions in performing different tasks related to the job give joy and satisfaction to the employees [39]. Security, the nature of the job, and amicable work conditions provide satisfaction and happiness to the workers [40,41]. Job satisfaction has a positive impact on the working attitudes of the employees [42]. A lack of satisfactory salary packages is a great hindrance to the job satisfaction of the employees [21].

The life satisfaction of library professionals also depends upon job satisfaction. A good job provides satisfactory living conditions [43]. Efficient leadership in academic libraries provides job satisfaction to the library workforce [44]. Effective human resource management (HRM) is a great tool in the provision of job satisfaction to library employees [45].

### 3.4. Job Performance of the Library Workforce

Job performance is related to work output which plays an essential role in the uplift of organizations [46]. It shows that workforces successfully complete assigned goals through modern-driven approaches [47].

Job security affects librarians’ performance a great deal in academic libraries so it has a significant relationship with job performance [48]. The facilities of university libraries are a great source for increasing the work performance of librarians [49]. Different human resource management styles affect the job performance of the library workforce. Therefore, the role of the management should be considered carefully for producing productive result outcomes for the academic library staff [50]. For the efficient functioning of academic libraries, the creative output of library professionals is of paramount significance [51,52].

### 3.5. Motivational Factors to Enhance Work Output and Satisfaction Level of Academic Librarians

State-of-the-art facilities provided to library staff in the universities enhance their satisfaction and job performance [9]. Motivation occurs in academic library staff through the provision of regular incentives from the policymakers of the institutions because such incentives and honorariums boost their morale [14].

The provision of adequate facilities during the job improves staff performance in university libraries [53,54]. The satisfaction of different needs of academic library staff including basic needs, physiological needs, esteem needs, and psychological needs is beneficial in improving performance [54]. Field trips, recreational activities, the availability of required tools, and balanced work hours create positive passion among library staff [55]. Service benefits need to be provided to the library staff so that they may carry out additional services for the prosperity of the universities [56].

The implementation of IT in university libraries is a constant factor of motivation for librarians to enhance job performance [55]. This revolution in the field of librarianship is a significant driver in motivating university library professionals to initiate user-centric services by adopting the latest technological tools [57,58].

Factors of self-interest, positive attitude towards the job, and passion to learn and improve are fruitful tools to enhance work performance [59]. Willpower and self-determination prove dynamic factors in motivating library staff [60]. The passion for self-development to lead in the field brings productive results for the universities [55].

The good attitude of the seniors, the fair distribution of rewards, the passion for team spirit, and comfortable job conditions are motivational factors to improve the job performance of the academic librarians of the universities [33,58]. Appreciation from the employers to the university library staff stimulates them to work dedicatedly to enhance productivity [31]. For organizational effectiveness; the economic and social conditions of the academic library staff should be improved [61,62].

### 3.6. Impact of Motivation on the Job Performance of Librarians

Babalola and Nwalo [63] noted that motivation develops the work output of the workforce. Gobari et al. [64] assumed that motivation is the most effective tool that inspires workers to perform well in return for awards, incentives, appreciation, and honors. Cherry [65] argued that motivation has a positive impact on job performance and it brings fruitful results to organizations. Nnaeto and Ndoh [66] mentioned that optimal efficiency in organizations may be achieved if the workforce is empowered and motivated through different methods. Otagburuagu [67] reported that rewards and recognitions inspired library staff to work for the institutions with a dedicated attitude. Kaluarachchi and Gamlath [68] found that enhanced motivation produced effective results for the organization.

Pawirosumarto et al. [69] mentioned that the dedicated attitude of the library had a positive impact on their job performance. The proliferation of information technologies had made library users digitally literate, so library professionals needed to implement the latest tools for the provision of smart library services. Kaba [70] claimed that personal autonomy positively affected the work productivity of university librarians. Khoreva and Wechtler [71] concluded that internal motivation guided library staff towards productive results in the organizations. Anasi [72] concluded through his study that there was a positive relationship between motivation and the fruitful job output of academic librarians. Performance was measured in terms of the implementation of emerging technologies in the libraries. Paais and Pattiruhu [73] investigated the effects of motivation on employees’ satisfaction and job performance. They concluded that organizational culture and work motivation were positively associated with job performance. They further highlighted that effective leadership played a fruitful role in increasing the satisfaction level of the employees with their jobs. Knezevic et al. [74] discussed the importance of human resources for attaining productive work performance. They showed that motivated and skilled employees were true assets as they played a vital role in uplifting their institutions. Campbell and Goulding [75] stated that intrinsically and extrinsically motivated library staff carry out creative performance in university libraries. Jovanovic et al. [76] revealed through an empirical investigation that the organizational environment had a great impact on the job satisfaction level of the employees. Therefore, a comfortable work atmosphere needed to be provided to attain optimum productivity.

### 3.7. Analysis of National Studies

Rigor studies focusing on the topic of the ‘impact of motivational factors upon job performance in university libraries’ have not been conducted. This area is still unexplored in the setting of Pakistan; however, very few related studies have been conducted on the topic. Bhatti and Qureshi [77] concluded in their study that library staff should be actively engaged in the decision-making process to develop motivation and a high level of satisfaction with the job. Zafar et al. [47] revealed through their research that job satisfaction was related to the inner happiness of the employees due to certain benefits provided to them by employers in return for excellent services at the workplace. According to them, job skills and work conditions play a great role in providing satisfaction to the library staff. In recent research conducted by Ahmad et al. [78], it was found that the satisfaction of the physiological needs of the library staff brought improvement in job outcomes and enhanced productivity and efficiency. Bashir et al. [79] surveyed the effects of motivation on job output in the telecommunication sector. They revealed that healthy work–life conditions provided motivation to the workforce, and they became dedicated towards the assigned duty. Comfortable work conditions and service benefits positively impacted staff efficiency. Job satisfaction played a positive role in increasing the work output of employees. Khurram and Khan [80] concluded through a systematic review study that motivation was a great force that changed the mindset of library staff and encouraged them to apply the latest technologies in their universities.

## 4. Research Hypotheses

**H1a.**  *Motivational factors have a significant positive impact on the job performance level of academic librarians.*

**H1b.**  *Motivational factors have a significant impact on the job satisfaction level of librarians.*

**H2a.**  *The expectation of benefits has a significant positive impact on the job outcomes of library professionals working in academic libraries.*

**H2b.**  *The expectation of benefits has a significant positive impact on the satisfaction level of librarians working in academic libraries.*

## 5. Theoretical Framework

**Figure 1:**
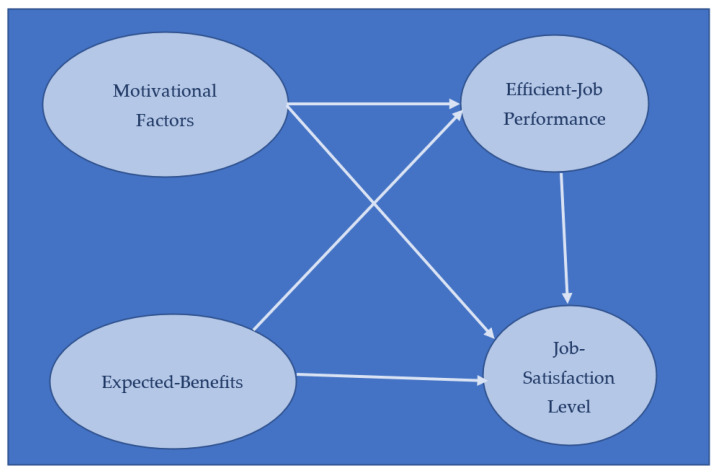
Theoretical framework of the study.

This study is based upon two theories known as the motivation theory and the expectancy theory. Hypotheses of the study have been developed through different constructs of these two theories. Motivation theory is related to internal elements which include self-inspiration, personal interest, and external elements which consist of incentives, rewards, and recognition. Expectancy theory is related to expectancy (effort), instrumentality (performance), and valence (reward). Authors shaped the theoretical framework (Figure 1) having used relevant constructs to test formulated hypotheses for finding out the impact of motivational factors on the job performance of academic librarians working in the university libraries of Lahore, Pakistan. The researchers used the variables of willpower, sense of honor, freedom, self-interest, and appreciation in the element of motivation. The variables of inner happiness, psychological ownership, professional learning opportunities, protection of rights, and institutional support were applied to the element of satisfaction, whereas in the element of performance, sub-categories consisting of satisfactory job output, efficient work outcomes, innovative work style, the implementation of emerging technologies, and increases in practical skills were used. The impact of independent variables on the dependent variables was measured through the application of inferential statistical tests.

## 6. Materials and Methods

A survey research method followed by predictive correlational design was applied to test the developed hypotheses in this study. Correlational research “is a research method that gives the researcher the opportunity to describe the relationship between two measured variables; whether two variables are correlated” [81]. This design facilitates researchers to make predictions from one variable to another [82].

The population of the study was university library professionals working in the university libraries of Lahore, Pakistan. There were 13 public sector universities and 21 private sector universities. The census sampling technique was used to collect data from the respondents. Library professionals with at least a Master’s degree were the participants of the study. Data were collected with the help of a questionnaire. The questionnaire was distributed among all library professionals working in these universities. The data of the working librarians were attained through professional directories and institutional websites.

The questionnaire was developed in light of a comprehensive literature analysis keeping in view the constructs of the already-developed hypotheses. A peer-review process was used to check the validity of the questionnaire. The tool was refined in light of feedback obtained by experts in the field. The reliability of the questionnaire was checked via a pilot testing process through the distribution of the questionnaire among 25 participants.

The questionnaire was distributed among 225 participants. Out of 225 respondents, 189 duly filled questionnaires were received. Hence, the response rate was 84%. The gathered data were analyzed through SPSS software. Descriptive tests of frequency, mean, standard deviation, and inferential statistical tests of regression were applied to find out the impact of motivation on the job outcomes of information professionals.

## 7. Data Analysis and Results

### 7.1. Demographic Information of the Respondents

The results of the study revealed that the majority of the participants (n = 147) were male and (n = 42) respondents were female (Table 1).

The findings showed that most of the respondents (n = 67) were aged between 31–35. In total, 49 respondents belonged to the age group 26–30. A total of 39 respondents were in the age group 36–40, 22 respondents were above 40 years old, and 16 respondents were aged between 20–25 (Table 1).

The majority of the respondents (n = 127) had an MLIS qualification. In total, 55 respondents had an M. Phil. degree in LIS, whereas only 7 respondents had earned the degree of Ph.D. (Table 1).

The results indicated that the majority of the participants (n = 106) were working in public sector universities and 83 respondents were employed in private sector universities. The findings of the study showed that the majority of the respondents (n = 54) had professional job experience of 1–5 years. A total of 42 respondents had professional work experience of 11–15 years. In total, 77 respondents had professional experience of 6–10 years, 36 respondents had experience of between 16 to 20 years, and 20 respondents had above 20 years’ experience (Table 1).

The results revealed that the majority of the respondents (n = 59) were librarians, 36 respondents were chief librarians, 31 respondents were senior librarians, 29 respondents were deputy librarians, 17 respondents were deputy chief librarians, and 17 were assistant librarians (Table 1).

### 7.2. Descriptive Analysis of Different Statements Related to the Study’s Key Variables

The findings of the study displayed that different variables of motivation impacted upon job productivity and satisfaction levels positively. These motivational factors included willpower, a sense of honor, freedom, self-interest, and appreciation. These factors had a positive relationship with job productivity and satisfaction levels according to the respondents’ opinions. The opinions of the participants were measured using different statements related to motivation, satisfaction, and job performance through descriptive analysis in SPSS software. A five-point Likert scale was used to measure mean and standard deviation regarding different statements about the key variables of the study. Most of the respondents agreed that motivational factors enhanced their job productivity. Table 2 shows mean responses with standard deviation received for different statements related to motivation, satisfaction, and performance. The top three mean scores were received for the statements, “Sense of honor leads to psychological ownership” (M = 4.29); “freedom of the employees protects rights”; “motivational factors move towards satisfactory job output”; “motivation is valuable in increasing the practical skills of the library staff” (M = 4.13), and “self-interest encourages the workforce”; “motivational drivers lead towards efficient work outcomes”; “motivational elements stimulate workers to adopt an innovative work style” (M = 4.11).

The study revealed that most of the respondents agreed that different elements provided them satisfaction, and they became more dedicated to the delivery of efficient library services. The elements of satisfaction included inner happiness, psychological ownership, professional learning opportunities, protection of rights, and institutional support. The results of the study showed that innovative job performance was carried out as a result of motivation and satisfaction with the job.

### 7.3. Impact of Motivational Factors on the Job Performance of University Librarians

A linear regression test was applied through SPSS software to find out the impact of motivational factors (IV) on the job performance (DV) of library professionals carrying out services in the university libraries of Punjab, Pakistan. Table 3 shows the results of linear regression analysis for the impact of motivation on the job performance of university librarians. The strength of the relationship is reflected through R^2^, which is 0.263. The beta value of the independent variable is 0.513, which shows a significant impact of the independent variable (motivational factors) upon the dependent variable (job performance). The results show that the Sig. value is *p* < 0.01. Therefore, it can be concluded that motivation has a strong relationship with job performance in academic libraries. Hence, H1a is not rejected.

### 7.4. Impact of Motivational Factors on the Job Satisfaction Level of University Librarians

A linear regression test was applied through SPSS software to find out the impact of motivational factors (IV) on the job satisfaction level (DV) of library professionals carrying out services in the university libraries of Lahore, Pakistan. Table 4 shows the results of linear regression analysis for the impact of motivational factors on the job satisfaction level of university librarians. The strength of the relationship is reflected through R^2^, which is 0.261. The beta value of the independent variable is 0.515, which shows a significant impact of the independent variable (motivational factors) upon the dependent variable (job satisfaction level). The results reveal that the Sig. value is *p* < 0.02. Therefore, it can be concluded that motivational factors have a strong relationship with the job satisfaction level of academic libraries. Hence, H1b is not rejected.

### 7.5. Impact of Expectation of Benefits on Work Performance of Academic Librarians of Pakistan

Through Statistical Package for Social Science software (Version 26.0), a linear regression statistical test was utilized to investigate the impact of expectation of benefits motivation (IV) upon the work performance (DV) of professional library staff working in the university libraries of Pakistan. Table 5 shows results for the impact of the expectation of benefits on the work outcome of university library professionals. The strength of the impact is shown through R^2^, which is 0.265. The beta value of the regressor variable is 0.515, which shows a significant impact of the independent variable (expectation of benefits) upon the response variable (work output). The results show that the Sig. value is *p* < 0.01. Therefore, it can be declared that the expectation of benefits has a strong impact on the work output of university librarians. Hence, H2a is not rejected.

### 7.6. Impact of Expectation of Benefits on the Job Satisfaction Level of Academic Librarians in Pakistan

SPSS software was utilized to apply a regression test to measure the impact of the expectation of benefits motivation (IV) on the job satisfaction level (DV) of professional library workforces in the universities. Table 6 presents the impact of the expectation of benefits on the job satisfaction level of university library professionals. R^2^ is 0.196, which shows the strength of the impact. The beta value of the independent variable is 0.442, which shows a significant impact of the independent variable (expectation of benefits) upon the measured variable (job satisfaction level). The results display that the Sig. value is *p* < 0.01. Therefore, it can be inferred that expectation of benefits has a powerful impact on the job satisfaction level of university library professionals. Hence, H2b is accepted based on the retrieved results of the scientific evidence.

## 8. Discussion

The findings of the study showed that motivation is a necessary predictor of innovative job outcomes and satisfaction with the job. Motivation is a necessary component for inspiring library workforces to provide user-centric services and to enhance their level of satisfaction with their duty. The results revealed that willpower, appreciation, self-interest, freedom, and a sense of honor have a significant positive impact on productive job output. Inner happiness, psychological ownership, professional learning opportunities, protection of rights, and institutional support provided satisfaction to the library staff, and they became more dedicated to compliance with their organizational tasks. The majority of the respondents agreed that satisfactory job output, the delivery of efficient work outcomes, the adoption of innovative workstyles, the implementation of emerging technologies, and increases in practical skills were highly essential in the present age. These results are similar to the findings of the studies investigated by Babalola and Nwalo; Gobari et al.; Nnaeto and Ndoh; Anasi; and Campbell and Goulding [63,64,66,72,75].

Library administration should meet the different needs of the staff members to meet organizational goals. It was indicated by Kaluarachchi and Gamlath; Pawirosumarto et al.; Kaba; and Khoreva and Wechtler [68,69,70,71] that motivated library staff carried out innovative job performance. Intrinsically and extrinsically demotivated library staff may not perform productive tasks enthusiastically. Different motivational factors have a significant positive effect on the job performance of academic librarians in universities. Library staff should be motivated well by the senior management of universities for the attainment of creative performance.

The results of the study have revealed that internally satisfied employees play a vital role in the uplift of institutions. Motivated and satisfied library staff members carry out services through modern-driven techniques and tools. They play a leading role in the organization and initiate customized services according to the changing needs of library users. Different motivational factors, including financial benefits, a positive workplace environment, comfortable work routines, job promotion opportunities, awards, special incentives, and transformative leadership boost the satisfaction level of librarians towards the job. Internally dissatisfied staff may not perform innovative services effectively and efficiently.

The findings of the study have shown that motivational and behavioral factors have a positive relationship with innovative job productivity. Willpower, appreciation, self-interest, freedom, and a sense of honor are important factors that motivate librarians to implement emerging technological tools in academic libraries. Motivation provides workforces to enhance their professional skill sets; therefore, skilled staff proves a great asset to their organizations. These results are consistent with the findings of the studies conducted by Paais and Pattiruhu [73]; Bashir et al. [79]; Knezevic et al. [74]; and Jovanovic et al. [76].

**Figure 2:**
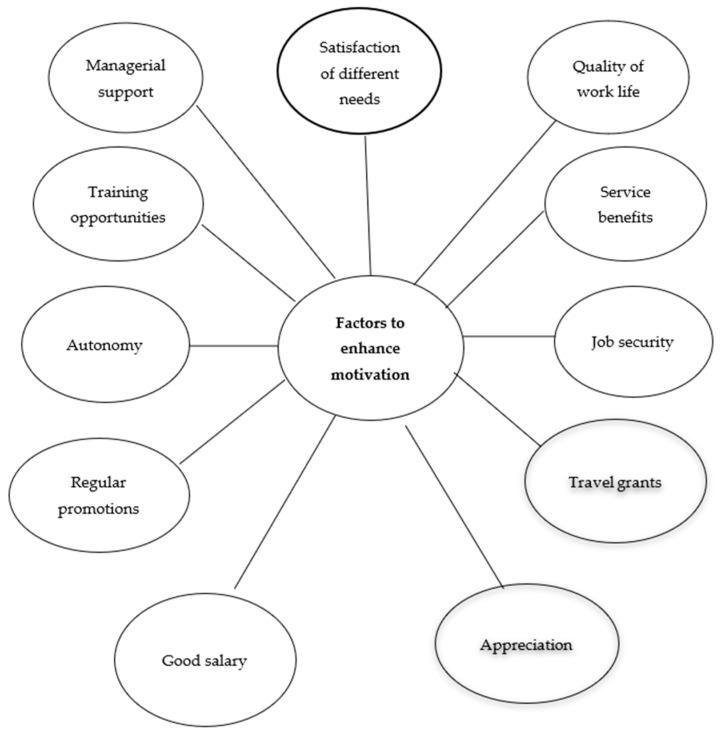
Framework to enhance the motivational level of academic librarians for innovative performance.

The study has used the human motivation theory of McClelland and Vroom’s expectancy theory for presenting a broader picture of the motivators affecting job productivity and the level of satisfaction of the library staff working in academic settings. Based on hypotheses tested through inferential statistical tests, the study has proposed a framework (Figure 2) for increasing the motivational level of library staff towards their jobs. Motivation and satisfaction are significant drivers in initiating user-centric services in university libraries. Table 7 provides an overview of the results of the investigation.

The provided framework (Figure 2) is very fruitful for policymakers, university administrations, head librarians, and other stakeholders as it has practical implications. Policymakers may use the given framework as a baseline to turn library staff into a motivated skilled workforce for the delivery of smart user services in academic institutions. Based on the findings of this study, a legal document may be developed by policymakers consisting of standardized protocols and policies, for enhancing the motivation and satisfaction level of librarians. Internal motivation may be imparted to librarians through continued professional development (CPD) opportunities. For external motivation, different incentives need to be offered. A documented policy duly approved by policymakers based upon core factors to motivate librarians to efficient job performance to uplift library services in institutions will provide a benchmark and guidelines for university administrators to take practical measures for creating motivation among library staff to enhance job satisfaction levels for innovative performance.

The investigation has shown that academic librarians should be motivated through different methods. Their needs should be satisfied. They should be empowered so that they might work with psychological ownership. The willpower of librarians should be increased through different training sessions. The workforce working in university libraries should be provided with appreciation, recognition, and rewards. The self-interest of librarians towards job tasks should be aroused. Library professionals should be provided autonomy to work independently with a sense of honor. Fruitful steps need to be taken to increase the inner happiness of library staff. The rights of the library workforce should be protected, and they should be provided institutional support in all circumstances. Motivation and satisfaction play a useful role in the provision of innovative job output, the delivery of efficient work outcomes, the adoption of exceptional workstyles, increases in practical expertise, and the implementation of emerging technologies. Educational institutions should take care of the different needs of their library employees so that they might implement value-added services to facilitate end-users effectively and efficiently.

## 9. Conclusions

Motivational factors have a significant positive impact on job performance and the satisfaction level of librarians. Different modes of motivation stimulate the human resource of the library to carry out productive services for the uplift of the parent organization. The expectation of benefits (time-scale promotions, best employee award, empowerment, comfortable job conditions, adequate salary package, state-of-the-art facilities, special incentives and honorariums, adequate counseling and coaching, job security, recreational activities, the availability of required tools, and service benefits) also plays a vital role in efficient work performance and increasing the job satisfaction level of library professionals performing services in universities. When workforces are provided benefits by organizers, productive outcomes are received, and the inner happiness of the employees also increases.

The study has contributed significant literature to the existing body of knowledge by testing the effects of motivational and behavioral factors on the job productivity of academic librarians in Pakistan through empirical investigation based upon a theoretical framework. Hypotheses were developed based on the constructs of two theories called the human motivation theory and expectancy theory of motivation. Based on the results of the tested hypotheses, it may be concluded that motivational factors have a positive impact on innovative job performance. Without motivated library staff, user-centric services may not be delivered in university libraries. The study has identified a positive relationship between motivational factors with job innovation among university librarians.

## 10. Implications of the Study

This research has theoretical implications for the researchers for further enhancing and modifying the theory of motivation. It also provides practical implications for policymakers and human resource managers to work on the motivation of their human resources for achieving efficient outcomes.

## 11. Limitations and Future Research

1. This study has been conducted through a predictive correlational research design. The respondents were not interviewed using a qualitative approach.
2. The current research provides numerical data obtained from only university librarians and not from college and school librarians.
3. The present study includes the population only from Lahore, Pakistan. Other cities have not been included due to a shortage of time and financial resources.
4. The study applied constructs from McClelland’s human motivation theory and Vroom’s expectancy theory.
5. The study’s findings may not be generalized to other parts of the world keeping in view the population of only one city’s universities in this study.

## Figures and Tables

**Table 1:** Descriptive analysis of demographic information of respondents (N = 189).

Variables		F	%
Gender			
	Male	147	77.78
	Female	42	22.22
Age (years)			
	20–25	16	8.46
	26–30	49	25.92
	31–35	67	35.45
	36–40	39	20.63
	>40	22	11.64
Qualification			
	MLIS	127	67.19
	M Phil/MS	55	29.10
	Ph.D.	7	3.70
Type of Institute			
	Public Sector	106	56.08
	Private Sector	83	43.91
Experience			
	1–5	54	28.57
	6–10	37	19.58
	11–15	42	22.22
	16–20	36	19.05
	Over 20	20	10.58
Designation			
	Chief Librarian	36	19.05
	Deputy Chief Librarian	17	8.99
	Deputy Librarian	29	15.34
	Senior Librarian	31	16.40
	Librarian	59	31.22
	Assistant Librarian	17	8.99

**Table 2:** Descriptive analysis of respondents’ opinions about different statements related to the study’s key variables (N = 189).

Motivation	Statements	Mean	Standard Deviation
	Will-power provides inner happiness.	3.24	0.778
	A sense of honor leads to psychological ownership.	4.29	0.819
	Freedom of the employees protects rights.	4.13	0.773
	Self-interest encourages the workforce.	4.11	0.871
	Appreciation motivates librarians to participate in professional learning activities.	3.07	0.880
	Motivational factors move towards satisfactory job output.	4.13	0.773
	Motivational drivers lead towards efficient work outcomes.	4.11	0.871
	Motivational elements stimulate workers to adopt an innovative work style.	4.11	0.871
	Motivation facilitates the implementation of emerging technologies	4.03	0.844
	Motivation is valuable in increasing the practical skills of the library staff.	4.13	0.773

Note (s): Scale used: strongly agree = 5, agree = 4, no opinion = 3, disagree= 2, strongly disagree = 1.

**Table 3:** Impact of motivational factors on job performance.

Model		Unstandardized Coefficients		Standardized Coefficients	T	Sig.
		B	Std. Error	Beta		
1	(Constant)	2.270	0.223		10.194	0.000
	Motivation	0.374	0.057	0.513	6.538	0.000

a. Dependent variable: work output. b. R^2^ = 0.263.

**Table 4:** Impact of motivational factors on job satisfaction level.

Model		Unstandardized Coefficients		Standardized Coefficients	T	Sig.
		B	Std. Error	Beta		
1	(Constant)	2.270	0.223		10.194	0.000
	Motivation	0.374	0.057	0.515	6.538	0.02

a. Dependent variable: work output. b. R^2^ = 0.261.

**Table 5:** Impact of expectation of benefits on work output.

Model		Unstandardized Coefficients		Standardized Coefficients	T	Sig.
		B	Std. Error	Beta		
1	(Constant)	2.270	0.223		10.194	0.000
	Expectation	0.374	0.057	0.515	6.538	0.000

a. Dependent variable: work output. b. R^2^ = 0.265.

**Table 6:** Impact of expectation of benefits on job satisfaction level.

Model		Unstandardized Coefficients		Standardized Coefficients	T	Sig.
		B	Std. Error	Beta		
1	(Constant)	2.435	0.243		10.006	0.000
	Expectation	0.335	0.063	0.442	5.359	0.000

a. Dependent variable: job satisfaction level. b. R^2^ = 0.196.

**Table 7:** Summary of hypotheses results.

Hypotheses	Beta Value	*p*-Value	Remarks
H1a	0.513	0.000	Accepted
H1b	0.515	0.002	Accepted
H2a	0.515	0.000	Accepted
H2b	0.442	0.000	Accepted

## Data Availability

The authors confirm that the data supporting the findings of this study are available within the article.
